# Percutaneous versus open posterior stabilization in AOSpine type A3 thoracolumbar fractures

**DOI:** 10.1186/s12891-020-3099-6

**Published:** 2020-02-05

**Authors:** Christoph J. Erichsen, Christoph-Eckhard Heyde, Christoph Josten, Oliver Gonschorek, Stephanie Panzer, Christian von Rüden, Ulrich J. Spiegl

**Affiliations:** 1 Department of Trauma Surgery, BG Trauma Center Murnau, Professor-Küntscher Str. 8, 82418 Murnau, Murnau am Staffelsee, Germany; 2 Department of Orthopaedics, Trauma Surgery and Reconstructive Surgery, University of Leipzig, Leipzig, Germany; 3 Department of Radiology, BG Trauma Center Murnau, Murnau am Staffelsee, Germany; 4 Institute for Biomechanics, Paracelsus Medical University, Salzburg, Austria

**Keywords:** Thoracolumbar fracture, Posterior open instrumentation, Additional anterior fusion, Percutaneous fixation, Sagittal balance

## Abstract

**Background:**

The purpose of this retrospective cohort study was to compare treatment strategies of two level-one trauma centers regarding clinical and radiological outcomes focusing on non-osteoporotic AOSpine type A3 fractures of the thoracolumbar spine at levels T11 to L2.

**Methods:**

Eighty-seven patients between 18 and 65 years of age that were treated operatively in either of two trauma centers were included. One treatment strategy includes open posterior stabilization whereas the other uses percutaneous posterior stabilization. Both included additional anterior fusion if necessary. Demographic data, McCormack classification, duration of surgery, hospital stay and further parameters were assessed. Owestry Disability Index (ODI), Visual Analog Scale (VAS) and SF-36 were measured for functional outcome. Bisegmental kyphosis angle, reduction loss and sagittal alignment parameters were assessed for radiological outcome. Follow up was at least 24 months.

**Results:**

There was no significant difference regarding our primary functional outcome parameter (ODI) between both groups. Regarding radiological outcome kyphosis angle at time of follow up did not show a significant difference. Reduction loss at time of follow up was moderate in both groups with a significantly lower rate in the percutaneously stabilized group. Surgery time was significantly shorter for posterior stabilization and anterior fusion in the percutaneous group. Time of hospital stay was equal for posterior stabilization but shorter for anterior fusion in the open stabilized group.

**Conclusion:**

Both treatment strategies are safe and effective showing only minor loss of reduction. Clinical relevant differences in functional and radiographic outcome between the two surgical groups could not be demonstrated.

**Trial registration:**

It was conducted according to ICMJE guidelines and has been retrospectively registered with the German Clinical Trials Registry (identification number: DRKS00015693, 07.11.2018).

## Introduction

Fractures of the thoracolumbar spine account for two-thirds of all spinal injuries [1]. These fractures are usually classified according to the AOSpine Classification [2]. Incomplete burst fractures (AOSpine type A3) represent the majority of thoracolumbar fractures [3]. When treated operatively management includes isolated anterior fusion, posterior stabilization using an internal fixation device in open or minimal invasive technique or combined posterior stabilization and anterior fusion [4–7]. During recent years, percutaneous minimally invasive internal stabilization of thoracolumbar spinal fractures without neurological disabilities has been established [8–13]. Nevertheless, there is still a controversy whether minimally invasive stabilization is superior to open posterior instrumentation. It remains unclear whether the minimal invasive technique is able to achieve sufficient fracture reduction and whether retention can be maintained.

Multiple earlier studies have evaluated radiological and clinical outcomes comparing both techniques [14–21] but none of them have focused on one specific fracture configuration and spinal localization.

Therefore, the aim of this study was to compare surgical treatment strategies of two level-one trauma centers regarding clinical and radiological outcomes focusing on isolated non-osteoporotic AOSpine type A3 fractures of the thoracolumbar spine at levels T11 to L2. We hypothesized that minimally invasive posterior stabilization achieves similar reduction rates as the open technique with lower complication rates.

## Patients and methods

### Study design

This study was designed as a retrospective cohort study. Patients between 18 and 65 years with traumatic incomplete burst fractures of the thoracolumbar junction (vertebral bodies T11 to L2) treated with either of the two treatment strategies at one of two level I trauma-centers from 2013 to 2015 were included. Treatment strategy in trauma center A included open posterior stabilization and additive anterior fusion if necessary. In trauma center B, minimally invasive posterior stabilization and additive anterior fusion if necessary was performed. Details of the two treatment strategies are described below.

Patients with other than AOSpine type A3 fractures, fractures older than four weeks, osteoporotic or pathologic fractures, neurological deficits, polytraumatized patients with an Injury Severity Score (ISS) ≥ 16, additional kyphoplasty or vertebroplasty, dorsal stabilization of more than two moving segments, pregnant patients, and those who were not able to give informed consent were excluded from the study.

### Demographic data

Gender, age, Body Mass Index (BMI), and mechanism of injury were documented in all cases. Fractures were classified according to the AOSpine classification [2] and additionally to the McCormack load sharing classification [22]. Localization of the fracture was assessed. Type of 360°- stabilization (posterior open versus minimally invasive fixation), additive video-assisted anterior thoracoscopic surgery (ATS), range of time between trauma and index surgery as well as between posterior to anterior stabilization, surgery time (posterior, anterior and implant removal), length of hospital stay for each surgical intervention, and complications were documented.

### Treatment strategies and surgical techniques

Treatment strategy in trauma center A was open dorsal fixation using the Universal Spine System (USS™; DePuy Synthes Companies, Oberdorf, Switzerland) and delayed (after 6 weeks) additive ATS (Obelisk™, Ulrich Medical, Ulm, Germany) in patients with McCormack Scores < 7 and without major disc herniation into the fracture zone. In patients aged 60 years or older (32%) additional cement augmentation of pedicle screws was used (PALACOS®, Heraeus Medical GmbH, Wehrheim, Germany). None of the patients in both trauma centers received additive kyphoplasty, vertebroplasty of the index level, or long segment instrumentation.

Treatment strategy in trauma center B included minimally invasive posterior fixation using the monoaxial (*n* = 40) or polyaxial (n = 4) Longitude System (CD Horizon Longitude™ Multi-Level Percutaneous Fixation System, Medtronic Spinal and Biologics Business, Memphis, TN, USA). In patients with Mc Cormack Scores ≥6 and in patients with signs of fracture associated disc pathology (vacuum sign in CT or MRI-pathology) received early (during first 2 weeks) additional ATS using the Tantalum cage (TM-S Cervical Fusion Device Trabecular Metal™ Technology, Zimmer Biomet, Warsaw, IN, USA) with or without additive MACS plate fixation (MACS TL® Modular Anterior Construct System for the Thoracic and Lumbar Spine, Aesculap, Tuttlingen, Germany). Percutaneous stabilizations have been controlled intraoperatively by 3D-scan (O-arm™ Surgical Imaging System, Medtronic Minimally Invasive Therapy Group, Minneapolis, MN, USA).

### Clinical outcomes

The Oswestry Disability Index (ODI) [23], patients’ pain level (VAS Score ranging from 0 to 10; 0: no pain; 10: worst pain) and SF-36 were assessed by questionnaires after written informed consent was given. ODI was determined as primary functional outcome parameter. It is a specific instrument for back pain including ten questions on limitations of daily living. A total calculated score of 0% represents the best health status, while 100% represents the worst [23]. The SF-36 questionnaire was used to assess the general health status. Results are given as a Physical Component Summary (PCS) and a mental dimension, represented by the Mental Component Summary (MCS) [24].

### Radiological outcomes

Bisegmental kyphosis angle was defined as primary radiological outcome parameter. It was measured from the upper end plate of the upper-instrumented vertebra to the bottom end plate of the lower-instrumented vertebra [25]. Anterior-posterior and lateral view radiographs of the thoracolumbar spine were performed preoperatively and postoperatively in standing position. Follow-up was performed at least two years after trauma including anterior-posterior and complete lateral spine views. Achieved reduction of kyphosis angle by operation and potential loss of reduction at time of follow-up was calculated.

As a secondary outcome parameter sagittal spine alignment was determined at time of follow-up [26, 27]. Thoracal kyphosis (TK), lumbar lordosis (LL), pelvic incidence (PI), pelvic tilt (PT), sacral slope (SS) and C7-lot were measured independently by two senior orthopedic surgeons and by one senior radiologist. Radiological images were evaluated using approved diagnostic monitors and Agfa IMPAX software (IMPAX EE, Agfa HealthCare, Bonn, Germany).

### Ethics approval

The study was approved by the institutional ethics committee of the Medical School of the University of Leipzig (approval number: 276/16-ek); it was conducted according to ICMJE guidelines, and has been retrospectively registered with the German Clinical Trials Registry (identification number: DRKS00015693). Written informed consent was obtained from all patients.

### Source of funding

This study was not funded.

### Statistical analysis

Statistical analysis was performed using statistical software R (RStudio Inc., Boston, MA, USA). Results in this study are presented as mean values with standard deviation (SD). Inductive analyses using the Wilcoxon rank-sum test and the Wilcoxon signed-rank test were performed to detect differences between the groups. A result was considered to be statistically significant with *p*-value < 0.05.

## Results

In total, 87 patients met the inclusion criteria between January 2013 and December 2015. Fourty-three patients underwent posterior open stabilization (OS; trauma center A) whereas the remaining 44 patients were treated using dorsal minimally invasive stabilization (MIS; trauma center B). Thirty-nine out of 44 patients treated with minimal invasive technique received monoaxial screws, four patients obtained polyaxial screws, and one patient a combination of both. Twenty-four patients in the OS group and 25 in the MIS group received additional anterior fusion. In trauma center A 23% of anterior fusions were peformed bisegmentally, whereas in trauma center B all patients were fused monosegmentally.

In trauma center A, timepoint of anterior fusion was 79 days (median) after posterior stabilization whereas in trauma center B it was seven days (median).

Regarding the McCormack load sharing classification, patients in the MIS group scored mostly 5 or 6 points (30 and 39%) while patients in the OS group scored 6 to 7 points (42 and 35%), without a significant difference. All patients in both groups scored at least 5 points. Both groups were comparable regarding gender, age and other demographic and clinical data with a significant difference regarding BMI (*p* = 0.0053; Table 1).

**Table 1:** Overview on patients` demographic data

	MIS				OS				
	n	%	Mean	Standard Deviation	n	%	Mean	Standard Deviation	*p*-value
Number of Patients	44				43				
Sex									0.1676
Male	19	43.2			26	60.5			
Female	25	56.8			17	39.5			
Age (years)	44		43.5	14.3	43		48.4	12.2	0.131
BMI	44		24.7	4.0	43		27.4	4.7	0.0053
Fracture localisation									0.1728
T11	2	4.5			1	2.3			
T12	14	31.8			15	34.9			
L1	24	54.5			22	51.2			
L2	4	9.1			5	11.6			
McCormack									0.267
3	0	0.0			0	0.0			
4	0	0.0			0	0.0			
5	13	29.5			6	14.0			
6	17	38.6			18	41.9			
7	8	18.2			15	34.9			
8	6	13.6			3	7.0			
9	0	0.0			0	0.0			
Mechanism of injury									
Fall < 3 m	15	34.1			20	46.5			
Fall > 3 m	6	13.6			10	23.3			
Car	7	15.9			5	11.6			
Motorcycle	2	4.5			1	2.3			
Bicycle					4	9.3			
Pedestrian vs. car					1	2.3			
Horse riding	4	9.1			1	2.3			
Paragliding	4	9.1							
Skiing	6	13.6							
Other					1	2.3			
Stabilization									
Monoaxial	39	88.6							
Polyaxial	4	9.1							
Combination	1	2.3							
Screw augmentation (yes)	0	0.0			14	32.6			
Implant removal (yes)	37	84.1			13	30.2			
ATS (yes)	25	56.8			24	55.8			
ATS bisegmental	0	0.0			10	23.3			

### Functional outcomes

**Fig. 1:**
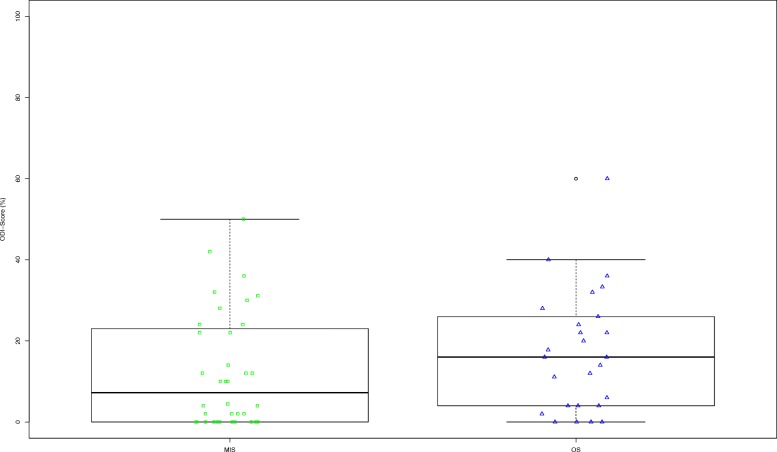
Boxplot ODI Score MIS versus OS at follow-up

In total, 62 out of 87 patients (71%) answered the questionnaires. There was no significant difference regarding ODI Score (MIS 12.3%, OS 18.3%) (Fig.1) or the PCS Component of SF-36 Score. The MCS Component of the SF-36 score showed a significant difference (*p* < 0.0001) with a lower score in the MIS group (41.9 points) than in the OS group (51.4 points). Furthermore, the MIS group showed a significantly lower VAS Score than the OS group (*p* < 0.0001; Table 2).

**Table 2:** Functional outcome

	MIS		OS		
	Mean	Standard Deviation	Mean	Standard Deviation	*p-*value
*ODI*					
ODI (%)	12.3	14.1	17.3	15.0	0.0619
VAS	1.7	1.9	3.1	1.7	0.0006
*SF-36*					
PCS	47.0	8.3	40.0	11.9	0.9863
MCS	41.9	7.1	51.4	12.6	< 0.0001

*ODI* Owestry Disability Index, *VAS* visual analog scale, *PCS* Physical component score, *MCS* mental component score

### Radiological outcomes

**Fig. 2:**
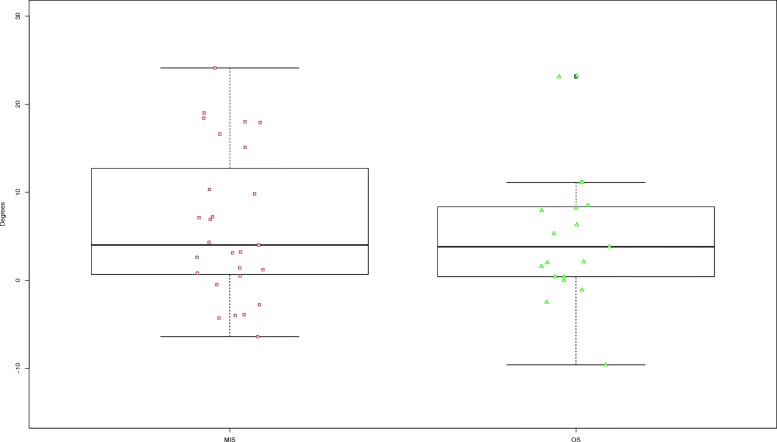
Boxplot Kyphosis angle MIS versus OS at follow-up

**Fig. 3:**
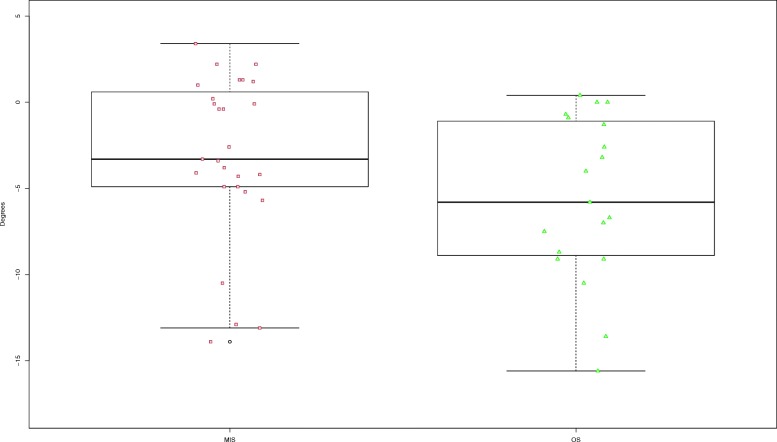
Boxplot Loss of reduction MIS versus OS at follow-up

In total 46 out of 87 patients (53%) participated on radiological follow-up at least 24 months after final surgery. Both techniques, MIS and OS achieved significant reduction of kyphosis angle preoperatively versus postoperatively (*p* < 0.001). The kyphosis angle at time of follow-up defined as primary radiological outcome parameter did not show a significant difference between the groups (*p* = 0.588; Fig.2). In both groups there was loss of reduction comparing imaging postoperatively and during follow-up. In the MIS group loss of reduction was 3.2°, in the OS group 5.6° showing a significant difference (*p* = 0.035; Fig.3).

The subgroup analysis of the patients in the MIS group that received additional anterior fusion (*n* = 14) revealed significantly less loss of reduction compared to those with posterior stabilization only (*n* = 13) (*p* = 0.025).

On the contrary, in the OS group (*n* = 13 versus *n* = 6) a statistical significant difference could not be detected (*p* = 0.059).

Regarding sagittal alignment parameters (SS, PI, PT, LL, TK and C7 Lot) no statistically significant difference between the two techniques could be revealed (Table 3).

**Table 3:** Radiological outcome

	MIS		OS		
	Mean	Standard Deviation	Mean	Standard Deviation	*p*-value
Kyphosis angle (°)	6.3	8.5	5.4	8.1	0.5883
Loss of reduction (°)	−3.2	4.8	−5.6	4.8	0.0353
PI	56.0	11.5	59.3	14.6	0.4515
PT	17.1	5.5	17.1	6.0	0.9446
SS	38.9	9.8	42.2	11.5	0.4870
LL	−51.5	10.6	− 47.2	13.1	0.5546
TK	42.9	8.6	39.4	7.5	0.1788
C7 Lot	3.9	2.9	15.5	29.5	0.1186

*PI* Pelvic Incidence, *PT* Pelvic Tilt, *SS* Sacral Slope, *LL* Lumbar Lordosis, *TK* Thoracal Kyphosis

### Operation time and hospital stay

The average range of time between trauma and surgery was 2.5 days in the MIS group and 3.6 days in the OS group, demonstrating a significant difference (*p* = 0.0023).

The duration of surgery was significantly shorter for posterior stabilization, anterior fusion, and implant removal in the MIS group compared to the OS group.

Hospital stay for patients who received posterior stabilization only showed no significant differences. Hospital stay for second surgery was significantly shorter in the OS group (*p* < 0.0001), but there was no significant difference for implant removal (Table 4).

**Table 4:** Duration of surgery and hospital stay

	MIS			OS			
*Duration of*	n	Mean	Standard Deviation	n	Mean	Standard Deviation	*p*-value
trauma to surgery (days)	44	2.5	4.0	43	3.6	3.0	0.0023
posterior stabilization (min)	44	63.5	22.3	43	106.7	31.1	< 0.0001
first hospital stay (days)	19	11.3	8.4	19	11.0	4.7	0.7039
first to second surgery (days)	25	23.6	49.3	24	120.2	125.8	< 0.0001
ATS (min)	25	99.5	18.7	24	136.5	39.0	0.0002
second hospital stay (days)	25	17.5	4.1	24	8.6	3.3	< 0.0001
implant removal (min)	37	33.5	8.0	13	49.2	11.5	< 0.0001
third hospital stay (days)	37	3.7	1.8	13	3.7	1.3	0.4056
until implant removal (days)	37	270.9	135.1	13	424.4	143.9	0.0004

### Complications

In the MIS group there was one suboptimal pedicle screw positioning where placement was closer to the endplate than desired without endplate affection. Therefore, no revision surgery was indicated. In one case drainage of pleural effusion after ATS was necessary.

In the OS group one case with pedicle screw loosening occurred. During scheduled ATS surgery these screws were replaced by screws with thicker diameter. One patient suffered from early postoperative wound infection and needed revision surgery. Implant removal was not necessary.

## Discussion

The focus of this study was to compare clinical and radiological results following posterior open or minimally invasive percutaneous thoracolumbar spinal fracture fixation. In contrast to earlier studies our work focuses on one specific fracture type (AOSpine type A3) limited to the thoracolumbar junction (T11 to L2) [14, 16–18] .

Both treatment groups in this study were comparable in terms of age, gender and other demographic data, fracture level as well as surgical technique and fixation materials. Regarding fracture pattern the OS group included more fractures of a higher McCormack score than the MIS group without any significant difference.

Regarding functional outcome, the ODI Score which was defined as our primary functional outcome parameter did not demonstrate significant differences between both groups. Our results only demonstrated a significant difference in the MCS Score of the SF-36 and in the VAS Score with lower scores in the MIS group.

Bisegmental kyphosis angle was defined as our primary radiological outcome parameter. In terms of achieved reduction analyzing kyphosis angle pre- and postoperatively both techniques revealed good reduction without any significant difference.

Loss of reduction at time of follow-up at least 24 months after initial surgery demonstrated a significant difference between open and percutaneous techniques.

In the subgroup that received additional anterior fusion the MIS group demonstrated significantly less loss of reduction compared to the OS group. These results have to be interpreted carefully due to low patient numbers and minimal differences in terms of *p*-values.

The sagittal alignment parameters (PI, PT, SS, LL, TK and C7-Lot) were comparable in both groups demonstrating no significant difference.

The duration of surgery was significantly lower for posterior stabilization, anterior fusion, and implant removal in the MIS group compared to the OS group. Multiple other studies also reported significantly shorter surgery times for the percutaneous technique [8, 17, 18]. The comparably less extensive surgical approach in the minimally invasive technique may reduce operation time for primary stabilization and for implant removal.

Summarizing our main findings, a major difference comparing the open versus the percutaneous approach could not be demonstrated in the current study.

Data comparing functional outcomes of both techniques is rare due to different patient populations and operative approaches. Pishnamaz et al. included 43 patients with open and 29 with percutaneous posterior stabilization of fractures of the thoracolumbar spine. The majority were AOSpine type A3 (*n* = 29) fractures, but also type A2, A4, and B1 fractures were found. They did not detect significant differences in radiological or functional outcome comparing the open to the percutaneous approach [18]. Fitschen-Oestern et al. compared 104 patients between the age of 15 and 86 including vertebral bodies T9-L3 as well as fracture classifications of all types. In the subgroup with A3 fractures (*n* = 35) they could not find significant differences in postoperative kyphosis angle between minimally invasive and open posterior stabilization. Functional outcome was not evaluated [17].

In contrast to open procedures, surgical tools for secure reconstruction of the physiological alignment of the thoracolumbar spine are limited using percutaneous techniques. In this study, both techniques obtained significant reduction of kyphosis angle preoperatively versus postoperatively. In the MIS group, this could be achieved mainly by optimized positioning of the patient including ventral sagging and lifting of the upper body. Using the percutaneous technique, reconstruction of the physiological spinal kyphosis in our patient group was sufficient in contrast to comparable earlier trials e. g. provided by Grass et al. 2006 who reported a relatively high amount of cases with incomplete vertebral body reduction [8]. The difference might be related to improved surgical instruments and methods during recent decades. Especially the use of monoaxial screw systems and stiffer chrome-cobalt rods have led to more stable constructs as biomechanical studies have demonstrated [28, 29]. The consistent use of monoaxial screws and tools is therefore recommended as mandatory precondition for precise percutaneous reduction of posttraumatic kyphosis after thoracolumbar burst fractures [12].

Our data demonstrated a moderate loss of reduction level in both groups with a significant difference (− 3.2° vs. -5.6°). Within the subgroups the patients that received open posterior stabilization and additional anterior fusion did not show a difference to those who received open posterior stabilization only. However, patients who received the percutaneous technique and additional anterior fusion had significantly less loss of reduction than those with dorsal percutaneous stabilization only (1.0° vs. 5.6°). These findings suggest that in indicated cases additional anterior fusion should be performed to support the posteriorly achieved reduction. There is a controversial debate about the best timepoint to perform additional anterior fusion. Spiegl et al. reported that in patients that received posterior monoaxial stabilization and additional delayed anterior fusion after 6 weeks did not lead to more reduction loss [30]. On the other hand, Sander et al. reported a comparably high rate of traumatic vertebral disc lesions 1 year after trauma without any disc pathology in the initial MRI following trauma [31]. Thus, delayed MRI might be more sensitive in terms of identifying traumatic vertebral disc lesions. Thereby, delayed anterior fusion aims to reduce the number of anterior fusions. This concept was performed in trauma center A in patients without indirect signs of vertebral disc lesions and McCormack scores lower than 7 [30]. Therefore, the period between posterior stabilization and additional anterior fusion was significantly longer in trauma center A.

An advantage of early additional anterior fusion might be the lower surgical effort due to less sclerosis and therefore easier surgical preparation when performed at an earlier timepoint. This is supported by the significantly lower surgery time in our data (99.5 vs. 136.5 min). Especially for patients that are working early anterior fusion might have economic advantages because return to professional life is sooner than in delayed anterior fusion. Disadvantages include that some patients who might have been stable enough with posterior stabilization only possibly received an unnecessary additional anterior fusion when operated at an earlier point of time.

Reported disadvantages of percutaneous posterior stabilization include higher rates of radiation exposure and difficulty to control fracture reduction and to maintain lordosis [8, 32, 33]. Advantages of the percutaneous technique such as protection of autochthonous back muscles, less blood loss, shorter operation time, lower risk of infection, shorter duration of hospital treatment, less postoperative pain levels, earlier pain relief and improved clinical outcomes have been reported in literature [14, 34–36]. Our data supports many of these findings. As mentioned above we also found a significantly shorter surgery time for the MIS group (63.5 min versus 106.7 min). Surprisingly, the current study revealed equal duration of hospital stay for patients who received posterior stabilization only. Pishnamaz et al. [18] also reported almost equal lengths of hospital stay while Fitschen-Oestern et al. [17] reported shorter times for the percutaneous technique. Other reported advantages such as less blood loss have not been evaluated in this study. Suction devices are rarely used for the percutaneous technique making it difficult to assess intraoperative blood loss.

Malpositioning of pedicle screws in thoracolumbar spinal fracture fixation is not avoidable completely but considered to be safe also in percutaneous techniques [37]. In this study, one pedicle screw was placed too close to the vertebral disc in the MIS group, but no surgical revision was necessary due to malpositioning of pedicle screws in both groups. The majority of percutaneous stabilizations have been controlled intraoperatively by 3D-scan. This as well as surgical experience may explain the good positioning of pedicle screws in the percutaneous technique in our data set.

### Study limitations

We acknowledge some limitations of the present study. First of all, this was a retrospective study design with all its limitations. Furthermore, duration of intraoperative radiological imaging and blood loss have not been assessed. Previous studies have shown that radiological imaging in minimally invasive stabilization may be more extensive and blood loss significantly less [8, 33, 34, 36]. Focusing on one specific fracture configuration limited to vertebral bodies T11 to L2 lead to smaller patient groups even though the study was performed at two high-volume level I trauma centers, but higher numbers of included patients would have been desirable.

## Conclusion

Both treatment strategies are equally safe and effective in terms of achieved reduction, loss of reduction, functional outcome and complication rates. Loss of reduction was relatively low in both treatment groups. Relevant differences in clinical and radiographic outcome between the two surgical groups could not be demonstrated.

## Data Availability

The datasets used and/or analysed during the current study are available from the corresponding author on reasonable request.

## Abbreviations

ATS: Video-assisted anterior thoracoscopic surgery
BMI: Body mass index
CT: Computed tomography
ISS: Injury severity score
LL: Lumbar lordosis
MCS: Mental component summary
MIS: Minimally invasive stabilization
MRI: Magnetic resonance imaging
ODI: Oswestry disability index
OS: Open stabilization
PCS: Physical component summary
PI: Pelvic incidence
PT: Pelvic tilt
SS: Sacral slope
TK: Thoracal kyphosis
USA: United States of America
USS: Universal spine system
VAS Score: Visual analog scale
